# Quality of Life in Patients with Hidradenitis Suppurativa: A Scoping Review

**DOI:** 10.3390/nursrep16080289

**Published:** 2026-08-19

**Authors:** Francesca Gambalunga, Viviana Lora, Cristina Marzo, Simona Molinaro, Flavia Pantaleo, Roberto Latina, Tatiana Bolgeo, Federica Dellafiore, Fabrizio Petrone, Nicolò Panattoni, Laura Iacorossi

**Affiliations:** 1 Professional Health Care Services Department, University Hospital “Policlinico Umberto I”, 00161 Rome, Italy; f.gambalunga@policlinicoumberto1.it; 2 Department of Clinical Dermatology, San Gallicano Dermatological Institute, IRCCS, 00144 Rome, Italy; viviana.lora@ifo.it; 3 Department of Biomedicine and Prevention, University of Rome Tor Vergata, 00133 Rome, Italy; simona.molinaro@ifo.it; 4 Nursing Research Unit IFO, IRCCS Regina Elena National Cancer Institute, Via Elio Chianesi 53, 00144 Rome, Italy; nicolo.panattoni@ifo.it; 5 Department of Life, Health and Health Professions Sciences, Link Campus University, 00165 Rome, Italy; f.pantaleo@unilink.it (F.P.); f.dellafiore@unilink.it (F.D.); l.iacorossi@unilink.it (L.I.); 6 Department of Health Promotion Science, Maternal and Infant Care, Internal Medicine, and Medical Specialties (PROMISE), University of Palermo, 90127 Palermo, Italy; roberto.latina@unipa.it; 7 Department of Research and Innovation, Azienda Ospedaliero-Universitaria SS Antonio e Biagio e Cesare Arrigo, 15121 Alessandria, Italy; tbolgeo@ospedale.al.it; 8 Health Professions Unit, IRCCS Regina Elena National Cancer Institute, Via Elio Chianesi, 53, 00144 Rome, Italy; fabrizio.petrone@ifo.it

**Keywords:** hidradenitis suppurativa, quality of life, scoping review, patient-reported outcomes, dermatology, symptoms

## Abstract

**Background/Objectives**: Hidradenitis Suppurativa (HS) is a chronic inflammatory skin disease associated with a significant impairment in patients’ quality of life (QoL). However, evidence on QoL assessment in HS remains fragmented, with heterogeneous instruments and domains reported. This scoping review aimed to map the assessment of QoL in individuals with HS, identify the instruments used, and describe the domains explored. **Methods:** This scoping review was conducted in accordance with the Arksey and O’Malley framework, as refined by Levac et al. and the Joanna Briggs Institute. A systematic search of six databases was performed between November 2025 and March 2026 to identify studies reporting QoL or patient-reported outcomes (PROs) in individuals with HS. Data were extracted and analyzed using a narrative synthesis approach. **Results:** A total of 21 studies met the inclusion criteria. HS was consistently associated with substantial QoL impairment across multiple domains, including physical, psychological, social, sexual, and occupational aspects. Symptom burden was a major determinant of reduced QoL, particularly pain, pruritus, malodor, and discharge. A wide range of instruments was used, with the Dermatology Life Quality Index (DLQI) being the most frequently applied. However, most tools did not adequately capture the multidimensional impact of HS. The use of HS-specific instruments was limited, and most studies adopted cross-sectional designs. **Conclusions:** QoL in HS is markedly compromised, but current assessment approaches are inconsistent and incomplete. Further research using comprehensive, disease-specific instruments and longitudinal designs is needed to better capture disease burden and support patient-centered care.

## 1. Introduction

Hidradenitis suppurativa (HS) is a chronic, recurrent inflammatory skin disease characterized by painful nodules, abscesses, sinus tracts, and scarring, typically affecting intertriginous areas such as the axillae, groin, and anogenital region [1]. Lesions may rupture, producing malodorous discharge that contributes to pain, functional limitations, and marked psychosocial distress [2,3]. In Europe, the prevalence of HS ranges from 0.03% to 1%, with disease onset most commonly in early adulthood and a higher incidence among women [4,5,6,7].

The chronic, painful, and unpredictable course of HS, combined with the sensitive localization of lesions and the limited availability of curative treatments, results in a substantial disease burden. Previous studies have consistently shown that HS has a greater negative impact on quality of life (QoL) than many other chronic dermatological conditions [8,9,10]. Patients frequently report persistent pain, social stigmatization, emotional distress, impaired body image, difficulties in intimate relationships, and reduced work ability [11,12]. Psychiatric comorbidities, including depression and anxiety, are also more prevalent in individuals with HS compared with the general population [13,14].

Despite the recognized impact of HS on patients’ lives, assessing QoL remains challenging. A wide variety of instruments have been used across studies, resulting in fragmented and heterogeneous evidence. Generic dermatology-related measures, such as the Dermatology Life Quality Index (DLQI), are the most commonly applied tools; however, they may not adequately capture HS-specific features [15,16]. Although several HS-specific QoL instruments have been developed, many still present limitations related to validation, sensitivity, or recall periods, and their use in clinical research remains limited [17,18].

International initiatives, such as the Hidradenitis Suppurativa Core Outcomes Set (HISTORIC), have emphasized the importance of evaluating QoL through a multidimensional framework encompassing physical, psychological, social, emotional, and work-related domains [19]. Nevertheless, these dimensions have not yet been systematically mapped across the existing literature. Given the heterogeneity of study designs, outcomes, and assessment tools, this scoping review aimed to provide a comprehensive synthesis of how QoL has been assessed in patients living with HS, to identify the instruments used, describe the domains explored, and highlight the remaining gaps in the evidence base [10,20].

## 2. Materials and Methods

### 2.1. Design

This scoping review was conducted in accordance with established methodological frameworks for evidence mapping studies. The review followed the framework originally proposed by Arksey and O’Malley [21], as further refined by Levac et al. [22] and the methodological guidance provided by the Joanna Briggs Institute (JBI) [23,24]. Reporting followed the PRISMA extension for scoping reviews (PRISMA-ScR) [25].

In line with the scope of this review, both QoL/Health-Related Quality of Life (HRQoL) instruments and patient-reported outcome measures (PROs) focusing on specific symptoms were considered. QoL instruments were defined as measures capturing the multidimensional impact of HS on daily life. At the same time, symptom-specific PROs were included when they explicitly explored patient-reported experiences closely linked to QoL impairment. These two categories were analytically distinguished throughout the review. The review protocol was registered with the Open Science Framework (OSF) on 25 May 2026 (https://doi.org/10.17605/OSF.IO/AUEPJ) after initiation of the database searches.

No substantive deviations or amendments to the review objectives or eligibility principles were made after the search commenced. Clarifications and operational definitions introduced during the review process are reported transparently in Supplementary Table S1, together with their timing and rationale.

### 2.2. Eligibility Criteria

Eligibility criteria were structured according to the Population–Concept–Context (PCC) framework recommended for scoping reviews.

Population: Studies involving adults diagnosed with hidradenitis suppurativa (HS) were eligible. No restrictions were applied regarding sex, disease severity, treatment status, or clinical phenotype. Studies including mixed dermatological populations were eligible only when data relating specifically to participants with HS could be identified separately. Diagnostic criteria and disease-severity classifications, when reported, were extracted as study characteristics rather than used as eligibility criteria.

Concept: The review considered quality of life (QoL), health-related quality of life (HRQoL), and patient-reported outcomes (PROs) reflecting symptoms or experiences directly related to QoL impairment. Eligible studies were required to report QoL/HRQoL outcomes, PROs, or the application of instruments assessing these constructs in individuals with HS. Studies exclusively focused on instrument development, psychometric validation, translation, or cross-cultural adaptation were excluded from the primary evidence synthesis.

Context: No restrictions were applied regarding geographical location, healthcare setting, or care pathway. Studies conducted in hospital, outpatient, specialist dermatology, community, or multicentre settings were eligible. Treatment status was not used as an inclusion criterion; however, studies exclusively evaluating therapeutic efficacy without reporting eligible QoL or PROs were excluded.

Peer-reviewed quantitative, qualitative, and mixed-methods primary studies meeting the PCC criteria were eligible. Secondary reviews relevant to QoL assessment in HS were retained only as contextual sources and were not treated as independent evidence units for the synthesis of patient-level QoL or PRO findings. No restrictions were applied regarding publication year or language. Grey literature and non-empirical publications, including editorials and commentaries, were excluded.

No substantive changes were made to the predefined eligibility principles or scope of the review after the search commenced. Clarifications introduced during the review process concerned wording and operational definitions only and are reported in Supplementary Table S1.

### 2.3. Protocol Amendments and Deviations

The registered protocol was compared with the procedures implemented in the final review. No substantive amendments or deviations were made to the review objectives or underlying eligibility principles after the search commenced. Clarifications concerning wording and operational definitions were documented with their timing and rationale in Supplementary Table S1. No unreported changes to the review objectives were made.

### 2.4. Information Sources

A comprehensive literature search was conducted across six electronic databases: PubMed/MEDLINE (PubMed interface), Cochrane Library (Wiley Online Library), CINAHL, (EBSCOhost), Web of Science Core Collection (Clarivate), Scopus (Elsevier), and PsycInfo (Ovid). These databases were selected to capture biomedical, nursing, psychological, and health-related literature relevant to QoL and PRO assessment in HS. The final search in each database was performed on the following dates: PubMed, [18 March 2026]; Cochrane Library, [18 March 2026]; CINAHL, [18 March 2026]; Web of Science, [18 March 2026]; Scopus, [18 March 2026]; and PsycINFO, [18 March 2026], no restrictions were applied regarding study design, publication year, or language.

### 2.5. Search Strategy

**Figure 1:**
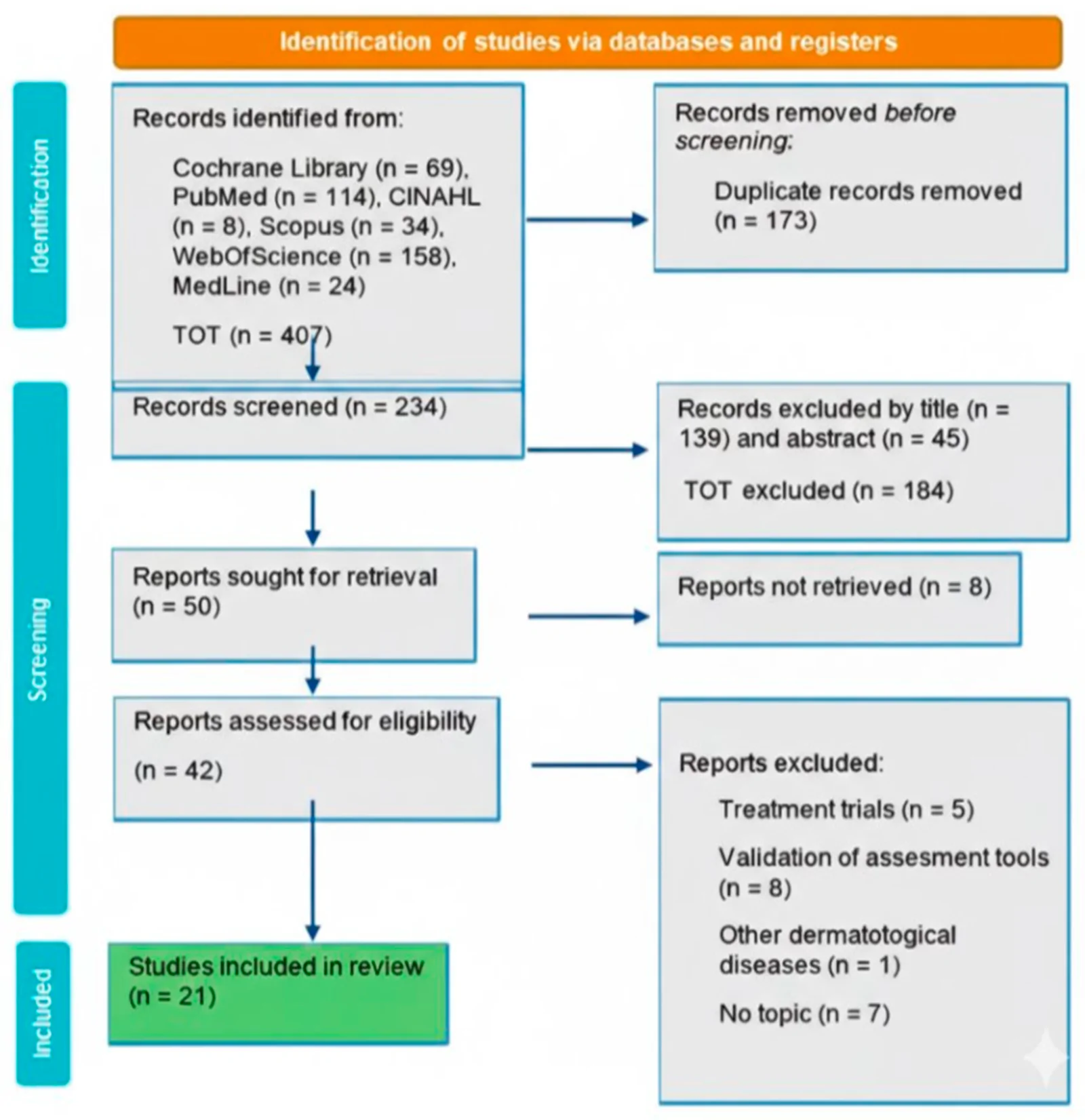
PRISMA flowchart illustrating the selection process and the number of sources included and excluded.

Search strategies were developed using a combination of Medical Subject Headings (MeSH), controlled vocabulary, and free-text terms related to HS, QoL, heath-related quality of life, and patient-reported outcomes. Syntax was adapted to the indexing system and interface of each database (Table 1). Reference lists of all included full-text reports and relevant secondary reviews were manually screened by two reviewers to identify additional potentially eligible studies. This process identified 234 additional records, of which 42 underwent full-text assessment and 21 were ultimately included. These records are reported separately in the PRISMA flow diagram (Figure 1).

### 2.6. Study Selection

Study selection was conducted in two stages. First, two reviewers independently screened titles and abstracts against the eligibility criteria. Records considered potentially eligible by either reviewer proceeded to full-text assessment. Full-text eligibility was independently assessed by two reviewers. Disagreements at either stage were first discussed between the reviewers; when consensus could not be reached, a third reviewer adjudicated.

### 2.7. Data Charting and Extraction

Data were charted using a structured form developed in Microsoft Excel, in line with JBI guidance [23]. The form was piloted on 21 included studies and refined before full extraction. Two reviewers independently extracted data, and discrepancies were resolved through discussion; unresolved disagreements were adjudicated by a third reviewer.Extracted data included author, year of publication, country, study design, study objectives, and the QoL or PRO instruments used.

### 2.8. Critical Appraisal of Individual Sources of Evidence

Consistent with the purpose of scoping reviews, no formal critical appraisal of individual studies was performed [26]. All eligible peer-reviewed studies were included to provide a comprehensive overview of the available evidence.

### 2.9. Synthesis of Results

A narrative synthesis was used to summarize and present the findings of this scoping review, consistent with established methodological frameworks for scoping studies [21]. Included studies were grouped according to whether they reported QoL outcomes or other patient-reported measures in patients with HS. QoL domains was developed iteratively during the synthesis. Domains explicitly reported by each study or assessment instrument were first extracted using the terminology of the original source. Conceptually related domains were subsequently grouped into broader descriptive categories: physical functioning and symptoms, psychological wellbeing, social relationships, sexual health, and occupational functioning. The grouping was intended solely to facilitate cross-study comparison and did not alter the validated structure or interpretation of the original instruments. Domain mapping was undertaken by two reviewers, with disagreements resolved through discussion and, where required, consultation with a third reviewer. This categorization was used to facilitate the interpretation of patterns across different assessment instruments and to describe the multidimensional impact of HS on patients’ quality of life. Findings are presented descriptively and supported, where appropriate, by tables and visual summaries to facilitate the interpretation of patterns across study designs, assessment instruments, and QoL domains.

Primary studies constituted the principal units of evidence for the synthesis of QoL and PRO findings. Systematic and narrative reviews were retained only to provide contextual information on measurement approaches, conceptual frameworks, and previously described evidence gaps. They were not treated as independent evidence units for patient-level findings. When a primary study was represented both directly in the review and within an included secondary review, the finding was synthesised from the original primary report only. Secondary reviews were not used to increase the frequency, weight, or apparent consistency of findings already represented by included primary studies. No formal weighting scheme was applied; primary evidence was prioritised, whereas secondary reviews had a contextual and interpretative role.

## 3. Results

### 3.1. Selection of Sources of Evidence

The search strategy yielded 407 records. After removal of duplicates, 234 unique citations were screened at the title and abstract level of which 184 were excluded. Full-text assessment was subsequently performed for 50 records. Of these, 8 reports could not be retrieved despite attempts to obtain the full texts, while 42 full-text reports were assessed for eligibility. A total of 21 studies met the eligibility criteria. The study selection process and reasons for exclusion at each stage are presented in the PRISMA flow diagram (Figure 1) [27].

Manual reference-list searching was also performed to identify additional potentially eligible studies. Individual bibliographic details and retrieval attempts for the 8 reports that could not be retrieved were not prospectively recorded and therefore could not be reliably reconstructed retrospectively.

Full-text reports excluded after eligibility assessment were not prospectively recorded with individual reasons for exclusion.

In accordance with scoping review methodology, secondary sources (systematic and narrative reviews) were included when relevant to QoL assessment, instruments, and frameworks, complement findings from primary research.

### 3.2. Characteristics of Sources of Evidence

**Figure 2:**
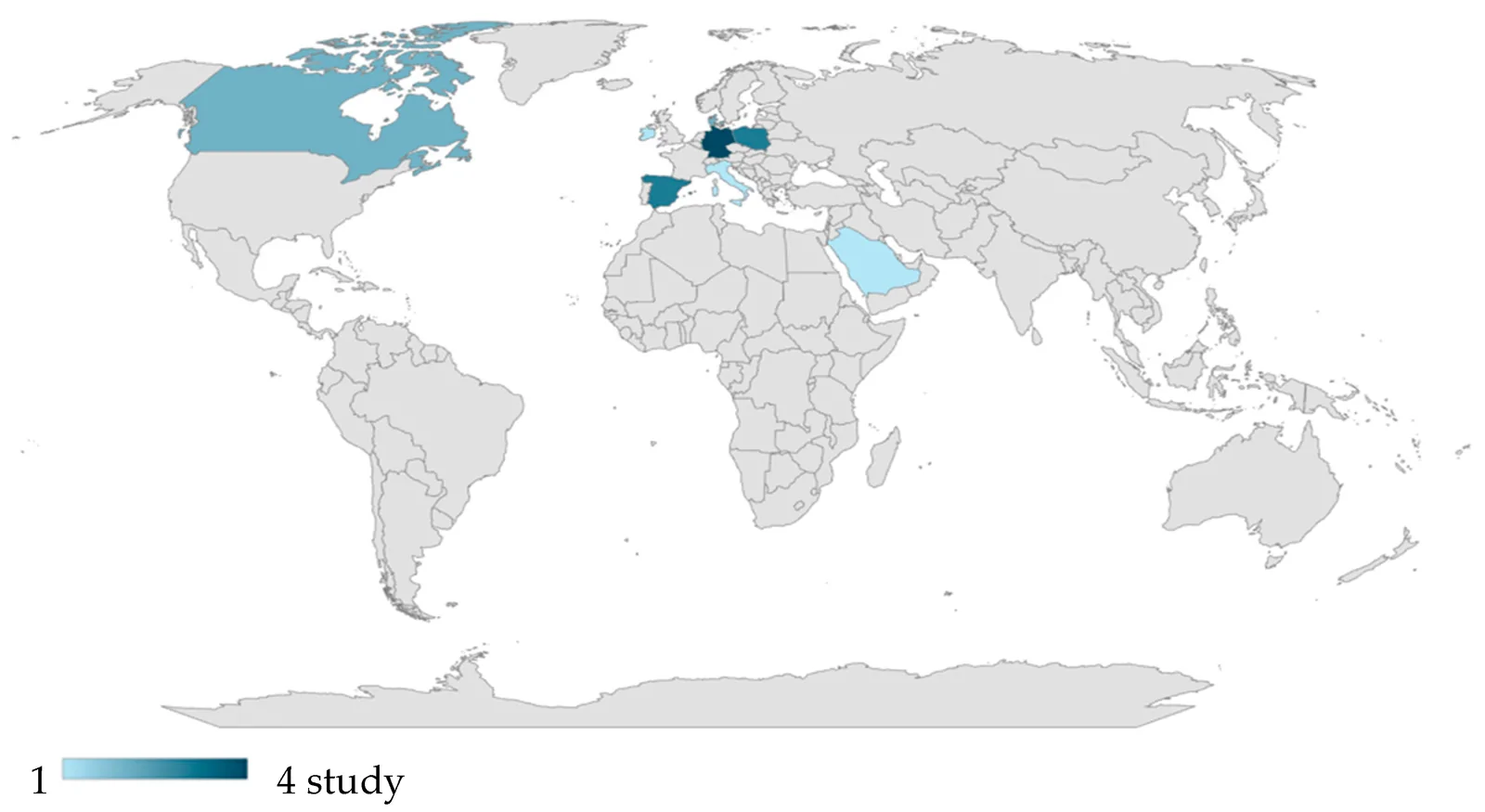
Map of included records per country.

The included studies were conducted across eight countries and spanned six geographical macro-regions. Most were carried out in Europe (*n* = 14, 67%), followed by North America (*n* = 2, 9.5%), the Middle East/Asia (*n* = 1, 5%), and international multicentre settings (*n* = 3, 9.5%). One study did not report the country of origin (*n* = 1, 5%). Germany was the most represented country, contributing four studies (19%) (Figure 2). Most articles were published in English (*n* = 20, 95%), and one in Spanish (*n* = 1, 5%).

### 3.3. Study Design and Temporal Distribution

**Figure 3:**
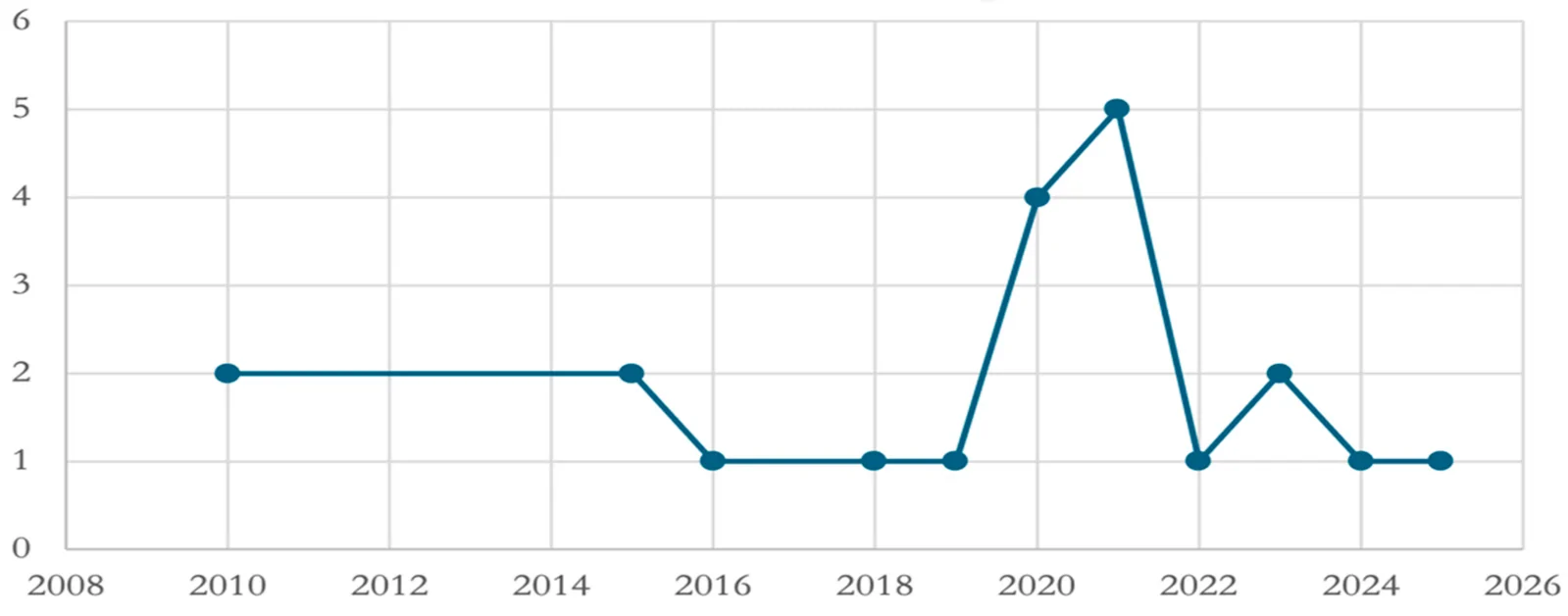
Number of included records per year of publication.

Publications increased over the past decade, peaking in 2021 (Figure 3). Most included studies were primary research (*n* = 15, 71.4%), with cross-sectional studies representing the largest group (*n* = 10, 47.6%). Other primary study designs included case–control, prospective observational, multicentre observational, and case-series studies.

Secondary research comprised six studies (29%), four systematic reviews (19%), and two narrative reviews (9.5%). Study designs and publication characteristics are detailed in Table 2.

### 3.4. Study Objectives

The objectives were heterogeneous and reflected the multidimensional burden of HS. Several studies aimed to assess the overall impact of HS on patients’ QoL and its association with clinical severity or demographic factors. Other studies focused on specific symptoms, most commonly pain, pruritus, malodor, and sleep disturbances, and their contribution to QoL impairment. Additional research explored psychosocial dimensions, including anxiety, depression, body image, sexual health, work productivity, and emotional burden. Finally, a subset of studies synthesised existing evidence or addressed the broader burden of HS, including diagnostic pathways and patient activation. The main characteristics of the included studies are summarised in Table 2.

### 3.5. Overview of QoL Assessment Approaches in Hidradenitis Suppurativa

Across the included studies, QoL and HRQoL in patients with HS were assessed using a heterogeneous range of validated patient-reported instruments (e.g., [28,29,30,31,32,33,34,35,36,37,38,39,40,41,42,43,45,46,47,48]). The tools employed varied considerably in scope, dimensionality, and disease specificity, reflecting the lack of a standardised approach to QoL assessment in HS.

Dermatology-specific instruments were the most commonly used tools, with the Dermatology Life Quality Index (DLQI) being applied in the majority of primary studies (e.g., [31,33,34,35,36,37,38,40,41,42,43,44,45,46,47]). Multidimensional dermatology-specific measures, such as Skindex-29 and Skindex-17, were used less frequently but allowed a broader evaluation of emotional, psychosocial, and symptom-related domains (e.g., [30,31,34,35,37]).

Generic HRQoL instruments, including the EQ-5D and the SF-36, were employed in a limited number of studies, mainly to assess overall health status and enable comparisons with the general population (e.g., [38,45,46]). Disease-specific QoL instruments for HS were used infrequently, indicating limited uptake of tailored tools in the existing literature (e.g., [32]).

An overview of the QoL and HRQoL instruments used across the included studies and the domains assessed is provided in Table 3. Overall, the different assessment approaches captured complementary but not fully comparable dimensions of HS burden. Dermatology-specific instruments, particularly the DLQI, provided a broad assessment of QoL impact but may not fully capture HS-specific concerns, whereas multidimensional and HS-specific tools allowed a more detailed evaluation of psychosocial and disease-related aspects. Generic HRQoL measures supported broader comparisons but appeared less sensitive to HS-specific symptom burden. These findings suggest that no single instrument fully reflects the multidimensional impact of HS and support the use of complementary assessment approaches. The QoL domains reported in this table correspond to those assessed by the original instruments and, where necessary, were grouped according to their validated domains.

### 3.6. QoL and HRQoL Instruments

Among the identified instrument, the DLQI was the most frequently used across the included studies and was applied to assess the impact of HS on patients’ daily lives [33,36,37,38,40,41,42,43,44,45,46,47]. Across studies, DLQI-based findings uniformly indicated a marked impairment in QoL. Several studies employed multidimensional dermatology-specific instruments, including Skindex-29 and Skindex-17 [30,31,34,35], which enabled a broader assessment of emotional, psychosocial, and symptom-related domains. These measures allowed a more detailed exploration of patients’ experiences compared with single-score instruments. Disease-specific QoL instruments for HS were used infrequently. Among these, the HS-specific QoL questionnaire was applied in a limited number of studies to assess the overall burden of the disease using a tailored approach [32]. Generic HRQoL instruments, such as the Short Form-36 (SF-36) and the EuroQol-5D (EQ-5D), were employed in a small subset of studies [38,45,46] to evaluate overall health status and health-related disutility, mainly to allow comparisons with the general population. Overall, the identified instruments captured different but partially overlapping dimensions of QoL impairment in HS. While dermatology-specific tools were useful for assessing the general impact of skin disease, multidimensional and HS-specific instruments provided greater insight into psychosocial and disease-related aspects that may remain underrepresented. These findings indicate that the choice of instrument influences the dimensions of HS burden identified and support the use of complementary assessment approaches. An overview of the QoL and HRQoL instruments used and the domains assessed across the included studies is presented in Table 3.

### 3.7. Symptom-Related Measures and Their Relationship with QoL

In addition to QoL instruments, several studies assessed key HS-related symptoms, using simple patient-reported scales [29,37,39]. Although these tools do not constitute QoL measures per se, the evidence consistently indicates that symptom burden plays a central role in driving QoL impairment. Pain emerged as the most influential symptom, followed by pruritus and malodor, which were frequently associated with emotional distress, social avoidance, and reduced daily functioning [33,40]. Sleep disturbance was also commonly reported and appeared closely linked to symptom severity [29,39].

### 3.8. Factors Associated with Poorer QoL

Across the included studies, several factors were consistently associated with poorer QoL in patients with HS. Higher disease severity and greater lesion burden were among the most frequently reported correlates of QoL impairment, although clinical severity scores did not fully explain patients’ perceived burden [36]. Lesion location also emerged as an important factor. Involvement of anogenital areas or other visible and sensitive body sites was associated with increased psychosocial distress and greater impairment in sexual function in [28,31,44]. Demographic and lifestyle factors, including female sex, higher body mass index, obesity, and smoking, were reported to be associated with worse QoL outcomes in several studies [36,37]. Psychological comorbidities, particularly anxiety and depression, frequently co-occurred with severe QoL impairment, highlighting the close relationship between emotional distress and the overall burden of HS [30,31].

### 3.9. QoL Domains Most Affected

Several QoL domains were consistently reported as being most affected in patients with HS. Physical functioning and daily activities were frequently impaired, largely due to pain, recurrent inflammation, and limitations in mobility and self-care [35,40,42]. Feelings of emotional distress, reduced self-esteem, and perceived stigma were repeatedly described, and often co-occurred with physical symptoms, amplifying overall QoL impairment [30,44]. Social relationships and sexual health emerged as particularly vulnerable domains. Difficulties in social interaction, intimacy, and sexual functioning were commonly reported and contributed to social withdrawal and impaired interpersonal relationships [28,31,44]. Work-related functioning represented another domain of substantial impact. Reduced work ability, absenteeism, and decreased productivity were frequently observed [35,40,42].

## 4. Discussion

This scoping review mapped the evidence on QoL and HRQoL among patients with HS, focusing on affected domains, assessment instruments, and literature gaps. Overall, the evidence depicts HS as a condition associated with a pervasive, multidimensional burden, across physical functioning, emotional well-being, social relationships, sexual health, and work-related functioning [28,35,48]. A central finding is the marked heterogeneity in QoL assessment approaches. Studies used a wide range of validated tools, with variability in scope, dimensionality, and disease specificity, limiting comparability [28,33,48]. Dermatology-specific instruments, most frequently the DLQI, were widely adopted, but may not fully capture HS-specific concerns [28,33,37]. The continued use of the DLQI in HS may reflect its ability to capture broader aspects of dermatological burden, as several of its domains overlap with areas affected by HS, including symptoms and feelings, daily activities, leisure, and interpersonal relationships. However, the specific impact of recurrent drainage, malodor, intimate-site involvement, and stigma may remain incompletely represented. The use of multidimensional dermatology-specific tools, such as Skindex-29 and Skindex-17, and domain-specific measures like the Body Image Quality of Life Inventory (BIQLI), suggests that single global instruments are insufficient to represent the lived experience of HS [30,31,34,44].

Although dermatology-specific instruments such as the DLQI remain the most frequently adopted measures, their widespread use should not obscure the growing relevance of disease-specific patient-reported outcome measures (PROMs). Generic and dermatology-specific questionnaires facilitate comparisons across dermatological conditions; however, they only partially capture several distinctive manifestations of hidradenitis suppurativa, including recurrent inflammatory flares, persistent pain, drainage, malodour, scarring, stigmatization, impairment of intimate relationships, occupational limitations, and the unpredictable course of the disease. These limitations have driven the development of HS-specific PROMs, which aim to provide a more accurate representation of the multidimensional burden experienced by patients while complementing, rather than replacing, generic quality-of-life instruments [49,52,53].

Among the currently available disease-specific instruments, the HSQoL-24 represents one of the most comprehensive questionnaires specifically designed for patients with HS. Developed through a patient-centred methodology, it comprises 24 items exploring psychosocial wellbeing, interpersonal relationships, occupational functioning, emotional health, economic burden and disease-related clinical limitations. By explicitly incorporating dimensions such as stigma, social isolation and the consequences of recurrent inflammatory episodes, HSQoL-24 addresses several aspects that remain underrepresented in instruments such as the DLQI and SF-36. Validation studies demonstrated satisfactory internal consistency, construct validity and reliability, supporting its application in both clinical practice and research. Furthermore, the availability of validated English, Polish and Serbian versions enhances its suitability for multicentre and cross-cultural investigations [49,50,51].

More recently, additional disease-specific instruments have further expanded the assessment of patient-reported outcomes in HS. The HiSQOL questionnaire was specifically developed to provide a concise yet multidimensional evaluation of disease burden while demonstrating responsiveness to changes in disease activity, making it particularly suitable for longitudinal observational studies and clinical trials [52]. Similarly, HIDRAdisk was conceived as a practical multidimensional visual instrument that facilitates communication between patients and healthcare professionals by integrating physical symptoms, psychological wellbeing, social functioning, sexuality, and work productivity into a single patient-centred assessment [53]. Although their use remains less widespread than that of traditional dermatology-specific questionnaires, these instruments provide clinically relevant information that complements existing measures and may improve the interpretation of treatment outcomes.

Generic HRQoL instruments, including the SF-36 and EQ-5D, were used in some studies to enable broader comparisons and estimate health-related disutility [38,45,46]. However, while useful for cross-condition comparisons, they appear less sensitive to the psychosocial and symptom-driven burden of HS, reinforcing the need for combined measurement strategies. Symptom burden consistently emerged as a key driver of QoL impairment. This suggests that patient-perceived disease burden may depend not only on inflammatory activity or lesion extent, but also on symptoms that directly interfere with everyday life, such as pain, drainage, malodour, and limitations in mobility or social participation. Therefore, symptom assessment should complement clinical severity measures when evaluating the impact of HS. These findings suggest that QoL in HS cannot be understood through clinical severity alone but must be considered alongside symptom experience and psychosocial context [33,36]. Mental health and psychosocial well-being appear central, with studies highlighting psychological distress, anxiety, depression, impaired self-esteem, and body image disruption as major correlates of QoL impairment [30,31,44]. Lesion location further modulates this burden, as involvement of anogenital or visible areas strongly affects sexuality and intimate relationships [28,31,44]. The disproportionate effect of anogenital involvement may be related to the intimate nature of these sites, where pain, drainage, and malodour can affect body image, sexual wellbeing, and interpersonal confidence, potentially leading to avoidance behaviours and social withdrawal. Work-related functioning is another consistently domain productivity, work limitations, and restrictions in daily activities commonly reported, underscoring the broader socioeconomic burden [35,40,42]. Occupational outcomes were often assessed alongside, rather than integrated within, QoL instruments, reflecting fragmentation in outcome measurement [35,42].

The complementary use of generic, dermatology-specific and disease-specific PROMs may therefore represent the most appropriate strategy for comprehensive quality-of-life assessment in hidradenitis suppurativa. Generic instruments such as the EQ-5D and SF-36 remain valuable for comparisons across diseases and for health-economic evaluations, whereas dermatology-specific questionnaires such as the DLQI ensure continuity with previous dermatological research. Conversely, HS-specific PROMs were developed to capture disease-relevant dimensions that may be incompletely represented by generic or dermatology-specific instruments, including recurrent inflammatory flares, malodour, drainage, stigma, intimate relationships and work productivity. Recent validation and cross-cultural adaptation studies have further strengthened the psychometric robustness and international applicability of HSQoL-24, supporting its broader implementation in multicentre observational studies and routine clinical practice [45,49,50,51]. Consequently, future studies should consider integrating generic, dermatology-specific and HS-specific instruments to achieve a more comprehensive and patient-centred evaluation of disease burden.

It should also be acknowledged that studies describing the development, psychometric validation and cross-cultural adaptation of HS-specific PROMs were intentionally excluded from the primary scoping review dataset because they did not meet the predefined eligibility criteria, which focused on studies applying QoL instruments rather than developing them. Nevertheless, these studies provide essential methodological context for interpreting the current evolution of quality-of-life assessment in hidradenitis suppurativa. Their discussion therefore aims to contextualise the findings of this review without modifying the scope or eligibility criteria originally defined in the review protocol [49,50,51,52,53].

This review also highlights important evidence gaps. Most studies were cross-sectional, limiting understanding of QoL trajectories over time and the impact of symptom fluctuations, disease progression, or care pathways [36,48]. Instrument variability further hampers comparisons across settings and populations, emphasizing the need for more consistent and HS-specific measurement strategies [32,33,34].

The identified gaps fall into three areas: (i) methodological gaps, including the predominance of cross-sectional designs and limited longitudinal evidence; (ii) measurement gaps, related to the heterogeneous and often non–HS-specific instruments used to assess QoL; and (iii) clinical gaps, reflecting the limited integration of psychosocial, sexual, and occupational domains into routine assessment strategies. These evidence gaps have important implications for clinical practice and future research. The lack of longitudinal evidence and the heterogeneity of QoL assessment approaches may limit the ability to monitor changes in patients’ needs over time and to evaluate the impact of interventions. In clinical practice, the integration of standardized QoL assessment alongside routine clinical evaluation may support a more comprehensive understanding of the burden of HS and promote patient-centered care. Considering the multidimensional impact of HS on physical symptoms, psychological wellbeing, social relationships, and occupational functioning, QoL assessment should be considered an important component of holistic disease management. In conclusion, HS substantially compromises QoL across multiple domains, with symptom burden and psychosocial distress playing a pivotal role. A patient-centered approach should integrate validated QoL instruments with structured symptom evaluation and attention to mental health, sexual wellbeing, and occupational functioning. Future research should prioritize longitudinal designs and improved standardization of outcome measures [28,33,35]. Although studies focused on the development and psychometric validation of disease-specific quality-of-life instruments were outside the predefined scope of this scoping review, HS-specific tools such as HSQoL-24 [49], HiSQOL [52], and HIDRAdisk [53] represent important advances in the assessment of quality of life in hidradenitis suppurativa. These instruments were developed to complement generic and dermatology-specific questionnaires by capturing disease features insufficiently represented by traditional measures, including pain, malodour, drainage, stigma, sexual impairment, and intimate-site involvement. Their greater responsiveness to clinical change and suitability for longitudinal monitoring, clinical trials, and registries make them increasingly relevant for contemporary HS research. Future studies should prioritize the broader implementation and cross-cultural validation of these instruments to enhance international comparability and support their integration into routine clinical practice. Their use in future clinical research and routine practice may facilitate a more comprehensive and disease-sensitive evaluation of patient-reported outcomes.

Overall, the progressive development of HS-specific PROMs reflects an important evolution towards more patient-centred outcome assessment. Future observational studies, disease registries and clinical trials should consider combining generic, dermatology-specific and HS-specific instruments to obtain a more comprehensive evaluation of disease burden, improve international comparability and facilitate the interpretation of patient-reported outcomes across different healthcare settings. Further validation studies involving diverse cultural and linguistic contexts will also contribute to the wider implementation of these instruments and promote greater standardisation of quality-of-life assessment in hidradenitis suppurativa [45,49,52,53].

### Limitations

This scoping review has several methodological limitations. First, the quality of included studies was not formally assessed. Although a formal critical appraisal was not performed, consistent with the mapping purpose of a scoping review, the available evidence showed considerable methodological heterogeneity, including differences in study design, sample characteristics, disease severity, care setting, and outcome assessment strategies. These factors should be considered when interpreting the breadth and generalizability of the findings. Second, heterogeneity of study designs, populations, and outcome measures, particularly about QoL instruments and symptom-specific tools, limited comparability and precluded quantitative synthesis. Third, the inclusion of secondary reviews as contextual sources introduced a potential risk of overlap with directly included primary studies. To minimise double counting, patient-level QoL and PRO findings were synthesised from original primary reports only, whereas secondary reviews were used exclusively to contextualise measurement approaches and broader evidence gaps. Nevertheless, some conceptual overlap between evidence sources cannot be completely excluded. Fourth, no substantive changes were made to the predefined Population, Concept, or Context eligibility criteria after the search commenced. Clarifications introduced during the review process concerned wording and operational definitions only and did not alter the review objectives or the predefined eligibility criteria. Fifth, studies exclusively focused on PROM development and psychometric validation were excluded from the formally included evidence set. Consequently, statements regarding the psychometric properties, responsiveness, and cross-cultural applicability of HS-specific PROMs rely on contextual validation literature rather than on the primary evidence synthesis. Sixth, the exclusion of treatment trials may have limited the capture of longitudinal QoL data and treatment-related changes in patient-reported outcomes. Future reviews may consider integrating interventional evidence to provide a more comprehensive evaluation of treatment-related QoL trajectories in HS. Seventh, although the search strategy was comprehensive and no language restrictions were applied, it was primarily developed using English-language terminology, which may have limited the retrieval of studies published in other languages. In addition, eight full-text reports could not be retrieved despite attempts to obtain the full texts, and relevant evidence may therefore have been missed. Finally, the predominance of cross-sectional studies limits interpretation of temporal trends and prevents causal inferences between disease severity, symptoms, and QoL outcomes. Despite these limitations, this review provides a structured overview of the available evidence and highlights important gaps for future research.

## 5. Conclusions

This scoping review shows that HS has a substantial impact on QoL across multiple domains. QoL impairment is driven not only by disease severity but also by symptom burden, psychosocial distress, and the lesion location. The findings highlight marked heterogeneity in the instruments used to assess QoL and related outcomes. Dermatology-specific tools are widely used but often fail to capture HS-specific concerns. HS-specific QoL instruments remain underutilized, and most evidence comes from cross-sectional studies, limiting insight into changes over time. Overall, the results underscore the need for more comprehensive and standardized approaches to QoL assessment in HS. Improved integration of multidimensional and HS-specific measures, together with longitudinal study designs, would strengthen the evidence base and better reflect patients’ lived experiences.

Future approaches to QoL assessment in HS should increasingly incorporate disease-specific patient-reported outcome measures, which may provide a more accurate representation of the multidimensional burden of the disease compared with generic instruments alone. The integration of HS-specific tools into clinical trials, disease registries, and routine clinical practice may improve the evaluation of treatment outcomes, facilitate longitudinal monitoring of patients, enhance the comparability of QoL outcomes across international studies through standardized HS-specific assessment, and support more personalized and patient-centred management strategies.

## 6. Implications for Nursing Practice

The findings of this scoping review highlight several implications for nursing and clinical practice. Given the strong association between symptom burden QoL impairment, routine symptom assessment using patient-reported measures may support a more comprehensive understanding of patients’ experiences. As clinical severity does not fully reflect the burden of HS, integrating QoL and PRO measures into clinical care may help identify individual needs beyond disease activity. These findings also emphasize the importance of patient education to promote treatment adherence, self-management, and informed decision-making. Furthermore, routine assessment of psychosocial wellbeing may facilitate the early identification of emotional distress and the timely referral of patients to appropriate support services when needed. Overall, a holistic, multidisciplinary approach, where nurses contribute through symptom monitoring, patient education, emotional support, and care coordination, may improve patient management. The use of multidimensional tools may help capture disease complexity and guide care planning.

## Figures and Tables

**Table 1:** Queries with keywords, Boolean operators, and Mesh terms were used for each database.

Query	Database
(“Hidradenitis Suppurativa”[Mesh] OR “hidradenitis suppurativa”[Title/Abstract] OR “acne inversa”[Title/Abstract]) AND (“Quality of Life”[Mesh] OR “quality of life”[Title/Abstract] OR QoL[Title/Abstract] OR HRQoL[Title/Abstract] OR “health-related quality of life”[Title/Abstract] OR “life satisfaction”[Title/Abstract] OR “life quality”[Title/Abstract] OR well-being[Title/Abstract] OR wellness[Title/Abstract] OR “standard of living”[Title/Abstract] OR “daily functioning”[Title/Abstract] OR “routine activities”[Title/Abstract] OR “social functioning”[Title/Abstract] OR “functional status”[Title/Abstract] OR “HiSQOL”[Title/Abstract] OR “DLQI”[Title/Abstract] OR “Skindex”[Title/Abstract] OR “EQ-5D”[Title/Abstract] OR “SF-36”[Title/Abstract]) AND (“subjective”[Title/Abstract] OR “self-reported”[Title/Abstract] OR “patient-reported”[Title/Abstract])	PubMed/MEDLINE (PubMed interface)
([mh “Hidradenitis Suppurativa”] OR “hidradenitis suppurativa”:ti,ab OR “acne inversa”:ti,ab) AND ([mh “Quality of Life”] OR “quality of life”:ti,ab OR QoL:ti,ab OR HRQoL:ti,ab OR “health-related quality of life”:ti,ab OR “life satisfaction”:ti,ab OR “life quality”:ti,ab OR well-being:ti,ab OR wellness:ti,ab OR “standard of living”:ti,ab OR “daily functioning”:ti,ab OR “routine activities”:ti,ab OR “social functioning”:ti,ab OR “functional status”:ti,ab OR HiSQOL:ti,ab OR DLQI:ti,ab OR Skindex:ti,ab OR EQ-5D:ti,ab OR SF-36:ti,ab) AND (subjective:ti,ab OR self-reported:ti,ab OR patient-reported:ti,ab)	Cochrane library (Wiley Online Library)
((MH “Hidradenitis Suppurativa+”) OR (TI “hidradenitis suppurativa” OR AB “hidradenitis suppurativa”) OR (TI “acne inversa” OR AB “acne inversa”)) AND ((MH “Quality of Life+”) OR (TI “quality of life” OR AB “quality of life”) OR (TI QoL OR AB QoL) OR (TI HRQoL OR AB HRQoL) OR (TI “health-related quality of life” OR AB “health-related quality of life”) OR (TI “life satisfaction” OR AB “life satisfaction”) OR (TI “life quality” OR AB “life quality”) OR (TI well-being OR AB well-being) OR (TI wellness OR AB wellness) OR (TI “standard of living” OR AB “standard of living”) OR (TI “daily functioning” OR AB “daily functioning”) OR (TI “routine activities” OR AB “routine activities”) OR (TI “social functioning” OR AB “social functioning”) OR (TI “functional status” OR AB “functional status”) OR (TI HiSQOL OR AB HiSQOL) OR (TI DLQI OR AB DLQI) OR (TI Skindex OR AB Skindex) OR (TI EQ-5D OR AB EQ-5D) OR (TI SF-36 OR AB SF-36)) AND ((TI subjective OR AB subjective) OR (TI self-reported OR AB self-reported) OR (TI patient-reported OR AB patient-reported))	Cinahl (EBSCOhost)
(“Hidradenitis Suppurativa” OR “hidradenitis suppurativa” OR “acne inversa”) AND (“Quality of Life” OR “quality of life” OR QoL OR HRQoL OR “health-related quality of life” OR “life satisfaction” OR “life quality” OR well-being OR wellness OR “standard of living” OR “daily functioning” OR “routine activities” OR “social functioning” OR “functional status” OR HiSQOL OR DLQI OR Skindex OR EQ-5D OR SF-36) AND (subjective OR self-reported OR patient-reported)	WOS Core Collection (Clarivate)
(“Hidradenitis Suppurativa” OR “hidradenitis suppurativa” OR “acne inversa”) AND (“Quality of Life” OR “quality of life” OR QoL OR HRQoL OR “health-related quality of life” OR “life satisfaction” OR “life quality” OR well-being OR wellness OR “standard of living” OR “daily functioning” OR “routine activities” OR “social functioning” OR “functional status” OR HiSQOL OR DLQI OR Skindex OR EQ-5D OR SF-36) AND (subjective OR self-reported OR patient-reported)	Scopus (Elsevier)
(exp “Hidradenitis Suppurativa”/ OR “hidradenitis suppurativa”.ti,ab. OR “acne inversa”.ti,ab.) AND (exp “Quality of Life”/ OR “quality of life”.ti,ab. OR QoL.ti,ab. OR HRQoL.ti,ab. OR “health-related quality of life”.ti,ab. OR “life satisfaction”.ti,ab. OR “life quality”.ti,ab. OR well-being.ti,ab. OR wellness.ti,ab. OR “standard of living”.ti,ab. OR “daily functioning”.ti,ab. OR “routine activities”.ti,ab. OR “social functioning”.ti,ab. OR “functional status”.ti,ab. OR HiSQOL.ti,ab. OR DLQI.ti,ab. OR Skindex.ti,ab. OR EQ-5D.ti,ab. OR SF-36.ti,ab.) AND (subjective.ti,ab. OR self-reported.ti,ab. OR patient-reported.ti,ab.)	PsycInfo (Ovid)

**Table 2:** Characteristics of the included studies.

Author	Year	Country	Study Design	Objective
Gooderham & Papp [28]	2015	Canada	Narrative review	To summarize existing evidence on the impact of hidradenitis suppurativa on quality of life.
Kaaz et al. [29]	2018	Germany	Case–control observational study	To evaluate the impact of pain and pruritus on sleep quality in patients with HS.
Frings et al. [30]	2019	Germany	Prospective observational study	To examine psychological burden, including anxiety, depression, and body image concerns, in patients with HS.
Quinto et al. [31]	2021	Italy	Cross-sectional study	To assess the impact of HS on sexual health and its association with quality of life.
Kokolakis et al. [32]	2025	Multinational (Europe)	Cross-sectional multicenter study	To explore patient activation, diagnostic delay, and their relationship with quality of life in HS.
Montero-Vilchez et al. [33]	2021	Spain	Systematic review	To synthesize evidence on the impact of HS symptoms (pain, pruritus, malodor) on quality of life.
Galvañ-Mas et al. [34]	2022	Spain	Systematic review	To examine the biopsychosocial impact of HS and its implications for quality of life.
Schneider-Burrus et al. [35]	2023	Germany	Multicenter observational study	To evaluate work productivity impairment and functional limitations associated with HS.
Schneider-Burrus et al. [36]	2021	Germany	Cross-sectional study	To identify factors associated with quality-of-life impairment in patients with HS.
Molina-Leyva et al. [37]	2020	Spain	Cross-sectional study	To analyze the impact of pruritus and malodor on quality of life and clinical characteristics in HS.
Alavi et al. [38]	2015	Canada	Case series	To describe quality-of-life impairment and its relationship with clinical severity in HS.
Yeroushalmi et al. [39]	2023	USA	Systematic review	To examine the association between HS and sleep disturbances.
Matusiak et al. [40]	2010	Poland	Cross-sectional study	To evaluate the impact of HS on quality of life and work activity.
Alamri et al. [41]	2021	Saudi Arabia	Cross-sectional study	To assess quality of life and its association with disease severity and anatomical distribution.
Kimball et al. [42]	2020	USA	Multicenter observational study	To examine the impact of HS symptoms on quality of life and work productivity.
Krajewski et al. [43]	2021	Poland	Cross-sectional study	To evaluate quality of life and demographic or clinical factors associated with QoL impairment.
Andersen et al. [44]	2020	Denmark	Cross-sectional comparative study	To compare body-image-related quality of life in HS versus other dermatological conditions.
Kimball et al. [45]	2024	USA	Systematic review	To synthesize evidence on the overall burden of HS, including quality of life.
Riis et al. [46]	2016	Denmark	Cross-sectional study	To assess health-related quality of life and disutility in patients with HS.
Matusiak et al. [47]	2010	Poland	Cross-sectional study	To examine psychophysical burden and its impact on quality of life.
Mac Mahon et al. [48]	2020	UK	Narrative review	To synthesize evidence on HRQoL and patient-reported outcomes in HS.

**Table 3:** QoL/HRQoL instruments identified in the included studies and contextual overview of currently availa-ble HS-specific PROMs.

Author, Year	QoL/HRQoL Instrument(s)	QoL Domains Captured (as Reported)	Instrument Category	Main Strengths	Main limitations	Languages/Cross-Cultural Validation
Gooderham & Papp, 2015 [28]	DLQI	Global QoL; physical; emotional; social	Dermatology-specific	Widely validated, easy to administer, commonly used in dermatology	May not fully capture HS-specific burden such as malodour, drainage, stigma, and sexual impairment	Available in numerous translated and cross-culturally validated versions worldwide
Kaaz et al., 2018 [29]	DLQI	Physical symptoms; emotional	Dermatology-specific	Allows comparison across dermatological diseases; simple and widely accepted	Limited sensitivity to HS-specific symptoms and psychosocial consequences	Available in numerous translated and cross-culturally validated versions worldwide
Frings et al., 2019 [30]	DLQI; Sk-29; BIQLI	Psychological; body image; social	Dermatology-specific; Domain-specific	Multidimensional assessment; captures emotional and functional consequences beyond disease symptoms	DLQI and Skindex-29 are not HS-specific; BIQLI evaluates only body image-related aspects	DLQI: extensively translated and validated internationally. Skindex-29 and BIQLI: available in multiple translated and validated versions, although less widely implemented than DLQI
Quinto et al., 2021 [31]	Sk-17	Psychosocial; physical; sexuality	Dermatology-specific	Shorter multidimensional dermatology tool; easier clinical application	Not developed specifically for HS; limited ability to capture HS-specific concerns	Available in translated versions with cross-cultural validation in selected populations
Kokolakis et al., 2025 [32]	HSQoL	Global QoL	HS-specific	Specifically developed to assess the unique burden of hidradenitis suppurativa and to capture aspects that may be underrepresented in generic dermatology instruments	Limited use compared with generic dermatology tools; further evidence on international applicability and cross-cultural validation is needed	Validated in the original development population; translated and cross-culturally validated versions are available in selected languages/populations (where applicable)
Montero-Vilchez et al., 2021 [33]	DLQI	Physical symptoms; emotional	Dermatology-specific	Widely validated, easy to administer, commonly used in dermatology studies	May not fully capture HS-specific aspects such as malodour, drainage, stigma, sexual impairment, and recurrent inflammatory episodes	Available in numerous translated and cross-culturally validated versions worldwide
Galvañ-Mas et al., 2022 [34]	DLQI; Sk-29	Biopsychosocial; emotional; social	Dermatology-specific	Combined use allows assessment of both general dermatological impact and emotional/functional burden	Neither instrument is HS-specific; disease-specific symptoms may remain incompletely captured	DLQI: extensive international validation. Skindex-29: multiple translated and culturally adapted versions available
Schneider-Burrus et al., 2023 [35]	DLQI	Global QoL; functional	Dermatology-specific	Enables comparison with other dermatological diseases; simple and widely accepted	Limited sensitivity for HS-specific burden and psychosocial consequences	Available in numerous translated and cross-culturally validated versions worldwide
Schneider-Burrus et al., 2021 [36]	DLQI	Global QoL; emotional; physical	Dermatology-specific	Commonly used outcome measure in dermatology and HS studies	Does not specifically address pain, drainage, malodour, stigma, or sexual health	Available in numerous translated and cross-culturally validated versions worldwide
Molina-Leyva et al., 2020 [37]	DLQI	Physical symptoms; emotional	Dermatology-specific	Short and practical; facilitates comparison across studies	Limited HS-specific content	Available in numerous translated and cross-culturally validated versions worldwide
Alavi et al., 2015 [38]	DLQI; SF-36	Global QoL; physical; emotional	Dermatology-specific; Generic HRQoL	Allows assessment of both dermatological burden and general health status	Limited ability to capture HS-specific symptoms and stigma	DLQI and SF-36 have extensive international translation and cross-cultural validation
Yeroushalmi et al., 2023 [39]	DLQI	Physical; emotional (QoL)	Dermatology-specific	Widely recognized and easy to interpret	May underestimate HS-specific QoL impairment	Available in numerous translated and cross-culturally validated versions worldwide
Matusiak et al., 2010 [40]	DLQI	QoL; work; psychophysical	Dermatology-specific	Established dermatology QoL measure	Not designed to capture HS-specific burden	Available in numerous translated and cross-culturally validated versions worldwide
Alamri et al., 2021 [41]	DLQI	Global QoL; physical	Dermatology-specific	Simple, validated, widely used	Limited disease specificity	Available in numerous translated and cross-culturally validated versions worldwide
Krajewski et al., 2021 [43]	DLQI	Global QoL; severity associations	Dermatology-specific	Facilitates comparison among dermatological conditions	Does not fully capture HS-related psychosocial and symptom burden	Available in numerous translated and cross-culturally validated versions worldwide
Andersen et al., 2020 [44]	BIQLI	Body image; relational; emotional	Domain-specific	Specifically evaluates body image consequences, relevant in visible/chronic skin diseases	Does not assess the full multidimensional burden of HS	Available in translated versions with validation studies in selected populations
Riis et al., 2016 [46]	EQ-5D	HRQoL; disutility	Generic HRQoL	Useful for health-economic evaluations and comparisons across diseases	Limited sensitivity for HS-specific symptoms and psychosocial impact	Available in numerous languages with extensive international validation and use across healthcare settings
Kimball et al., 2024 [45]	DLQI; EQ-5D	Global QoL; HRQoL; disutility	Dermatology-specific; Generic HRQoL	Provides complementary information on dermatological impact and overall health utility	Neither tool is HS-specific	Both instruments have broad international translation and cross-cultural validation
Mac Mahon et al., 2020 [48]	DLQI; SF-36; EQ-5D	HRQoL; global QoL; PROs overview	Dermatology-specific; Generic HRQoL	Allows evaluation of QoL from different perspectives and comparison with other conditions	Generic measures may miss HS-specific symptoms and psychosocial burden	All instruments have extensive international validation and are available in multiple languages
Contextual overview of HS-specific PROMs (not included in the scoping review dataset)						
Pedraz et al., 2019 [49]	HSQoL-24	Psychosocial wellbeing; interpersonal relationships; occupational functioning; emotional wellbeing; economic burden; disease-related limitations	HS-specific	Comprehensive disease-specific questionnaire capturing dimensions insufficiently represented by generic dermatology instruments	Limited implementation in observational studies and clinical trials to date; responsiveness evidence remains comparatively limited	Originally developed in Spanish; subsequently translated and cross-culturally validated in English, Polish and Serbian [50,51].
Kirby et al., 2021 [52]	HiSQOL	Symptoms; psychosocial burden; daily functioning; emotional wellbeing	HS-specific	Patient-informed instrument; responsive to changes in disease activity; suitable for longitudinal assessment and clinical trials	Limited diffusion outside recent HS research; further international validation and routine-care implementation are needed	Developed and validated in English and Danish populations; additional translations require formal cross-cultural validation
Zouboulis et al., 2017 [53]	HIDRAdisk	Physical symptoms; psychological wellbeing; sexuality; work productivity; social functioning; daily activities	HS-specific	Visual multidimensional instrument facilitating patient-clinician communication and repeated assessment	Limited use in observational studies; fewer validated language versions; visual/electronic format may affect implementation	Validated Italian version; broader international and linguistic validation remains limited

Note. HSQoL-24, HiSQOL, and HIDRAdisk are presented as contextual measurement evidence and were not part of the formally included evidence set. Development, validation, translation, and cross-cultural adaptation studies were excluded from the primary review according to the predefined eligibility criteria. Statements concerning their measurement properties are therefore based on the cited psychometric literature and should not be interpreted as findings generated by the present scoping-review synthesis. Abbreviations: QoL = Quality of Life; HRQoL = Health-Related Quality of Life; DLQI = Dermatology Life Quality Index; Sk-29 = Skindex-29; Sk-17 = Skindex-17; HSQoL = Hidradenitis Suppurativa Quality of Life questionnaire; SF-36 = Short Form-36 Health Survey; EQ-5D = EuroQol 5-Dimensions questionnaire; BIQLI = Body Image Quality of Life Inventory.

## Data Availability

No new data were created or analyzed in this study. Data sharing does not apply to this article.

## Supplementary Materials

The following supporting information can be downloaded at: https://www.mdpi.com/article/10.3390/nursrep16080289/s1, File S1: Prisma-ScR Checklist; Table S1: Eligibility criteria comparison; Table S2: Complete search strategies; Table S3: Characteristics of the included primary studies; Table S4: Full-Text screening exclusions.

## Public Involvement Statement

No public involvement in any aspect of this research.

## Guidelines and Standards Statement

This manuscript was drafted in accordance with the Preferred Reporting Items for Systematic Reviews and Meta-Analyses extension for Scoping Reviews (PRISMA-ScR) guidelines for scoping review research.

## References

[1] Jemec G.B., Heidenheim M., Nielsen N.H. (1996). Hidradenitis suppurativa—Characteristics and consequences. Clin. Exp. Dermatol..

[2] Saunte D.M.L., Jemec G.B.E. (2017). Hidradenitis suppurativa: Advances in diagnosis and treatment. JAMA.

[3] Zouboulis C.C., Del Marmol V., Mrowietz U., Prens E.P., Tzellos T., Jemec G.B. (2015). Hidradenitis suppurativa/acne inversa: Criteria for diagnosis, severity assessment, classification and disease evaluation. Dermatology.

[4] Kirsten N., Petersen J., Hagenström K., Augustin M. (2020). Epidemiology of hidradenitis suppurativa in Germany: An observational cohort study based on a multisource approach. J. Eur. Acad. Dermatol. Venereol..

[5] Jemec G.B., Heidenheim M., Nielsen N.H. (1996). The prevalence of hidradenitis suppurativa and its potential precursor lesions. J. Am. Acad. Dermatol..

[6] Goldburg S.R., Strober B.E., Payette M.J. (2020). Hidradenitis suppurativa: Epidemiology, clinical presentation, and pathogenesis. J. Am. Acad. Dermatol..

[7] Garg A., Kirby J.S., Lavian J., Lin G., Strunk A. (2017). Sex-and age-adjusted population analysis of prevalence estimates for hidradenitis suppurativa in the United States. JAMA Dermatol..

[8] Esmann S., Jemec G.B. (2011). Psychosocial impact of hidradenitis suppurativa: A qualitative study. Acta Derm. Venereol..

[9] Nikolakis G., Kokolakis G., Kaleta K., Wolk K., Hunger R., Sabat R., Zouboulis C.C. (2021). Pathogenesis of hidradenitis suppurativa/acne inversa. Hautarzt.

[10] Wolkenstein P., Loundou A., Barrau K., Auquier P., Revuz J. (2007). Quality of Life Group of the French Society of Dermatology. Quality of life impairment in hidradenitis suppurativa: A study of 61 cases. J. Am. Acad. Dermatol..

[11] Kirby J.S. (2016). Qualitative study shows disease damage matters in hidradenitis suppurativa. J. Am. Acad. Dermatol..

[12] Schneider-Burrus S., Jost A., Peters E.M.J., Witte-Haendel E., Sterry W., Sabat R. (2018). Association of hidradenitis suppurativa with body image. JAMA Dermatol..

[13] Shavit E., Dreiher J., Freud T., Halevy S., Vinker S., Cohen A. (2015). Psychiatric comorbidities in 3207 patients with hidradenitis suppurativa. J. Eur. Acad. Dermatol. Venereol..

[14] Mavrogiorgou P., Juckel G., Reimelt A., Hessam S., Scholl L., Frajkur J.L., Stockfleth E., Bechara F.G. (2021). Psychiatrische komorbidität bei hidradenitis suppurativa/acne inversa. Hautarzt.

[15] Zouboulis C.C., Chernyshov P.V. (2021). Hidradenitis suppurativa-specific patient-reported outcomes measures. J. Eur. Acad. Dermatol. Venereol..

[16] Finlay A.Y., Khan G.K. (1994). Dermatology Life Quality Index (DLQI)—A simple practical measure for routine clinical use. Clin. Exp. Dermatol..

[17] Pinard J., Vleugels R.A., Joyce C., Merola J.F., Patel M. (2018). Hidradenitis suppurativa burden of disease tool: Pilot testing of a disease-specific QoL questionnaire. J. Am. Acad. Dermatol..

[18] Chiricozzi A., Bettoli V., De Pità O., Dini V., Fabbrocini G., Monfrecola G., Musumeci M., Parodi A., Sampogna F., Pennella A. (2019). HIDRAdisk: An innovative visual tool to assess the burden of hidradenitis suppurativa. J. Eur. Acad. Dermatol. Venereol..

[19] Thorlacius L., Ingram J.R., Villumsen B., Esmann S., Kirby J., Gottlieb A., Merola J., Dellavalle R., Nielsen S., Christensen R. (2018). A core domain set for hidradenitis suppurativa trial outcomes: An international Delphi process. Br. J. Dermatol..

[20] von der Werth J.M., Jemec G.B.E. (2001). Morbidity in patients with hidradenitis suppurativa. Br. J. Dermatol..

[21] Arksey H., O’Malley L. (2005). Scoping studies: Towards a methodological framework. Int. J. Soc. Res. Methodol..

[22] Levac D., Colquhoun H., O’Brien K.K. (2010). Scoping studies: Advancing the methodology. Implement. Sci..

[23] Peters M.D.J., Marnie C., Tricco A.C., Pollock D., Munn Z., Alexander L., McInerney P., Godfrey C.M., Khalil H. (2020). Updated methodological guidance for the conduct of scoping reviews. JBI Evid. Synth..

[24] Pollock D., Davies E.L., Peters M.D.J., Tricco A.C., Alexander L., McInerney P., Godfrey C.M., Khalil H., Munn Z. (2021). Undertaking a scoping review: A practical guide for nursing and midwifery students, clinicians, researchers, and academics. J. Adv. Nurs..

[25] Moher D., Liberati A., Tetzlaff J., Altman D.G., The PRISMA Group (2009). Preferred Reporting Items for Systematic Reviews and Meta-Analyses: The PRISMA statement. PLoS Med..

[26] Tricco A.C., Lillie E., Zarin W., O’Brien K.K., Colquhoun H., Levac D., Moher D., Peters M.D.J., Horsley T., Weeks L. (2018). PRISMA extension for scoping reviews (PRISMA-ScR): Checklist and explanation. Ann. Intern. Med..

[27] Page M.J., McKenzie J.E., Bossuyt P.M., Boutron I., Hoffmann T.C., Mulrow C.D., Shamseer L., Tetzlaff J.M., Akl E.A., Brennan S.E. (2020). The PRISMA 2020 statement: An updated guideline for reporting systematic reviews. BMJ.

[28] Gooderham M., Papp K. (2015). The psychosocial impact of hidradenitis suppurativa. J. Am. Acad. Dermatol..

[29] Kaaz K., Szepietowski J.C., Matusiak Ł. (2018). Influence of itch and pain on sleep quality in patients with hidradenitis suppurativa. Acta Derm. Venereol..

[30] Frings V.G., Bauer B., Glöditzsch M., Goebeler M., Presser D. (2019). Assessing the psychological burden of patients with hidradenitis suppurativa. Eur. J. Dermatol..

[31] Quinto R.M., Mastroeni S., Sampogna F., Fania L., Fusari R., Iani L., Abeni D. (2021). Sexuality in Persons with hidradenitis suppurativa: Factors associated with sexual desire and functioning impairment. Front. Psychiatry.

[32] Kokolakis G., Vilarrasa E., Garg A., Alarcon I., Go M., Newbold G., Mamareli A., Richardson C., McGrath B.M., Guillem P. (2025). Patient perspectives on the impact of living with hidradenitis suppurativa: Results from the global ‘HS uncovered’ burden of disease survey. J. Venom. Anim. Toxins Incl. Trop. Dis..

[33] Montero-Vilchez T., Diaz-Calvillo P., Rodriguez-Pozo J.A., Cuenca-Barrales C., Martinez-Lopez A., Arias-Santiago S., Molina-Leyva A. (2021). The burden of hidradenitis suppurativa signs and symptoms in quality of life: Systematic review and meta-analysis. Int. J. Environ. Res. Public Health.

[34] Galvañ Mas P.M., López-Casanova P., Verdú-Soriano J., Berenguer-Pérez M. (2022). ¿Afecta la hidradenitis supurativa a la calidad de vida del paciente? Una revisión de la literatura. Gerokomos.

[35] Schneider-Burrus S., Kalus S., Fritz B., Wolk K., Gomis-Kleindienst S., Sabat R. (2023). The impact of hidradenitis suppurativa on professional life. Br. J. Dermatol..

[36] Schneider-Burrus S., Tsaousi A., Barbus S., Huss-Marp J., Witte K., Wolk K., Fritz B., Sabat R. (2021). Features associated with quality of life impairment in hidradenitis suppurativa patients. Front. Med..

[37] Molina-Leyva A., Cuenca-Barrales C. (2020). Pruritus and malodour in patients with hidradenitis suppurativa: Impact on quality of life and clinical features associated with symptom severity. Dermatology.

[38] Alavi A., Anooshirvani N., Kim W.B., Coutts P., Sibbald R.G. (2015). Quality-of-life impairment in patients with hidradenitis suppurativa: A Canadian study. Am. J. Clin. Dermatol..

[39] Yeroushalmi S., Ildardashty A., Elhage K.G., Chung M., Bartholomew E., Hakimi M., Tahir P., Naik H.B., Bhutani T., Liao W. (2023). Hidradenitis suppurativa and sleep: A systematic review. Arch. Dermatol. Res..

[40] Matusiak Ł., Bieniek A., Szepietowski J.C. (2010). Hidradenitis suppurativa markedly decreases quality of life and professional activity. J. Am. Acad. Dermatol..

[41] Alamri A.M., Alzahrani A.A., Aldakhil A.M., E Alharbi H., A Yahya F. (2021). Quality of life of patients with hidradenitis suppurativa in Jeddah, Saudi Arabia. Cureus.

[42] Kimball A.B., Crowley J.J., Papp K., Calimlim B., Duan Y., Fleischer A., Sobell J. (2020). Baseline patient-reported outcomes from UNITE: An observational, international, multicentre registry to evaluate hidradenitis suppurativa in clinical practice. J. Eur. Acad. Dermatol. Venereol..

[43] Krajewski P.K., Matusiak Ł., von Stebut E., Schultheis M., Kirschner U., Nikolakis G., Szepietowski J.C. (2021). Quality-of-life impairment among patients with hidradenitis suppurativa: A cross-sectional study of 1795 patients. Life.

[44] Andersen P.L., Nielsen R.M., Sigsgaard V., Jemec G.B., Riis P.T. (2020). Body image quality of life in patients with hidradenitis suppurativa compared with other dermatological disorders. Acta Derm. Venereol..

[45] Kimball A.B., Kirby J., Ingram J.R., Tran T., Pansar I., Ciaravino V., Willems D., Lewis-Mikhael A.-M., Tongbram V., Garg A. (2024). Burden of hidradenitis suppurativa: A systematic literature review of patient reported outcomes. Dermatol. Ther..

[46] Riis P.T., Vinding G.R., Ring H.C., Jemec G.B. (2016). Disutility in patients with hidradenitis suppurativa: A cross-sectional study using EuroQoL-5D. Acta Derm. Venereol..

[47] Matusiak Ł., Bieniek A., Szepietowski J.C. (2010). Psychophysical aspects of hidradenitis suppurativa. Acta Derm. Venereol..

[48] Mac Mahon J., Kirthi S., Byrne N., O′GRady C., Tobin A. (2020). An update on health-related quality of life and patient-reported outcomes in hidradenitis suppurativa. Patient Relat. Outcome Meas..

[49] Hindelangk B., Aguieew J., Schwaez M., Bwewzhnoi A., Eyerich K., Ntziachristos V., Biedermann T., Darsow U. (2019). Non-invasive imaging in dermatology and the unique potential of raster-scan optoacoustic mesoscopy. J. Eur. Acad. Dermatol. Venereol..

[50] Krajewski P.K., Bardowska K., Matusiak Ł., Zepietowska M., Tyczyńska K., Marron S., Aragones L., Szepietowski J. (2022). Hidradenitis Suppurativa Quality of Life 24 (HSQoL-24) now available for Polish patients: Creation and validation of the Polish language version. Postep. Dermatol. Alergol..

[51] Marinković M., Stojičić M., Jović M.S., Rakocevic J., Bukumirić Z., Jurišić M., Jovanović M.D., Jeremić J., Vlahovic A.M., Vujčić I. (2026). Reliability and validity of the Serbian version of the Hidradenitis Suppurativa Quality of Life Questionnaire (HSQoL-24). J. Clin. Med..

[52] Kirby J.S., Thorlacius L., Villumsen B., Ingram J., Garg A., Christensen K., Butt M., Esmann S., Tan J., Jemec G. (2021). The Hidradenitis Suppurativa Quality of Life (HiSQOL) score: Development and validation of a measure for clinical trials. J. Am. Acad. Dermatol..

[53] Peris K., Lo Schiavo A., Fabbrocini G., Dini V., Patrizi A., Fusano M., Bianchi L., Guanziroli E., Guarneri C., Parodi A. (2019). HIDRAdisk: Validation of an innovative visual tool to assess the burden of hidradenitis suppurativa. J. Eur. Acad. Dermatol. Venereol..

