# Supplementary material for: Quality of Life in Patients with Hidradenitis Suppurativa: A Scoping Review
Source: Nurs Rep. 2026 Aug 19;16(8):289. doi: 10.3390/nursrep16080289 (PMC13516480; doi:10.3390/nursrep16080289)
Supplement: Supplementary file 1 [file nursrep-16-00289-s001.zip › Supplementary Table S1.pdf]

Supplementary Table S1: Eligibility criteria comparison

Criterion	Registered protocol	Revised Manuscript	Assessment
Population	Individuals/patients with hidradenitis suppurativa (HS)	Adults diagnosed with HS	Operational clarification
Age	Not explicitly specified	Adults	Eligibility clarification
Sex	Not explicitly specified	No restrictions regarding sex	Eligibility clarification
Disease Severity	Not explicitly specified	No restrictions regarding disease severity	Eligibility clarification
Treatment status	Not explicitly specified	No restrictions regarding treatment status	Eligibility clarification
Clinical phenotype	Not explicitly specified	No restrictions regarding clinical phenotype	Eligibility clarification
Diagnostic criteria	Not explicitly specified	No specific diagnostic classification system required; diagnostic criteria, when reported, were extracted as study characteristics	Operational clarification
Concept	QoL and PROs	QoL, HRQoL, and PROs reflecting symptoms or experiences directly related to QoL impairment	Operational clarification
Context/setting	Not explicitly specified	No restrictions regarding geographical location, healthcare setting, or care pathway	Eligibility clarification
Study design	Quantitative and qualitative studies	Quantitative, qualitative, and mixed-methods primary studies	Operational clarification
Publication year	No restriction	No restriction	No change
Language	No restriction	No restriction	No change
Publication type	Non-empirical publications excluded	Peer-reviewed primary studies eligible; grey literature and non-empirical publications excluded	Operational clarification
QoL/PRO measurement studies	Studies reporting QoL or PROs	Studies reporting QoL/HRQoL outcomes, PROs, or application of instruments assessing	Operational clarification

		these constructs; studies exclusively focused on instrument development, psychometric validation, translation, or cross-cultural adaptation excluded from the primary evidence synthesis	

Note: The comparison shows that no substantive changes were made to the underlying eligibility principles after the search commenced. Additional details in the revised manuscript represent operational clarifications intended to improve transparency and reproducibility.
