# Supplementary material for: Quality of Life in Patients with Hidradenitis Suppurativa: A Scoping Review
Source: Nurs Rep. 2026 Aug 19;16(8):289. doi: 10.3390/nursrep16080289 (PMC13516480; doi:10.3390/nursrep16080289)
Supplement: Supplementary file 1 [file nursrep-16-00289-s001.zip › Supplementary table S2.pdf]

Supplementary Table S2: Complete search strategies

Database	Platform/interface	Search date	Limits/filters	Records retrieved	Complete search strategy
**PubMed/MEDLINE**	PubMed	18 March 2026	None	114	(“Hidradenitis Suppurativa"[Mesh] OR "hidradenitis suppurativa"[Title/Abstract] OR "acne inversa"[Title/Abstract]) AND ("Quality of Life"[Mesh] OR "quality of life"[Title/Abstract] OR QoL[Title/Abstract] OR HRQoL[Title/Abstract] OR "health-related quality of life"[Title/Abstract] OR "life satisfaction"[Title/Abstract] OR "life quality"[Title/Abstract] OR well-being[Title/Abstract] OR wellness[Title/Abstract] OR "standard of living"[Title/Abstract] OR "daily functioning"[Title/Abstract] OR "routine activities"[Title/Abstract] OR "social functioning"[Title/Abstract] OR "functional status"[Title/Abstract] OR "HiSQOL"[Title/Abstract] OR "DLQI"[Title/Abstract] OR "Skindex"[Title/Abstract] OR "EQ-5D"[Title/Abstract] OR "SF-36"[Title/Abstract]) AND ("subjective"[Title/Abstract] OR "self-reported"[Title/Abstract] OR "patient-reported"[Title/Abstract])
**Cochrane Library**	Wiley Online Library	18 March 2026	None	69	([mh "Hidradenitis Suppurativa"] OR "hidradenitis suppurativa":ti,ab OR "acne inversa":ti,ab) AND<br>([mh "Quality of Life"] OR "quality of life":ti,ab OR QoL:ti,ab OR HRQoL:ti,ab OR "health-related quality of life":ti,ab OR "life satisfaction":ti,ab OR "life quality":ti,ab OR well-being:ti,ab OR wellness:ti,ab OR "standard of living":ti,ab OR "daily functioning":ti,ab OR "routine activities":ti,ab OR "social functioning":ti,ab OR "functional status":ti,ab OR HiSQOL:ti,ab OR DLQI:ti,ab OR Skindex:ti,ab OR EQ-5D:ti,ab OR SF-36:ti,ab) AND<br>(subjective:ti,ab OR self-reported:ti,ab OR patient-reported:ti,ab)
**CINAHL**	EBSCOhost	18 March 2026	None	8	((MH "Hidradenitis Suppurativa+") OR (TI "hidradenitis suppurativa" OR AB "hidradenitis suppurativa") OR (TI "acne inversa" OR AB "acne inversa")) AND<br>((MH "Quality of Life+") OR (TI "quality of life" OR AB "quality of life") OR (TI QoL OR AB QoL) OR (TI HRQoL OR AB HRQoL) OR (TI "health-related quality of life" OR AB "health-related quality of life") OR (TI "life satisfaction" OR AB "life satisfaction") OR (TI "life quality" OR AB "life quality") OR (TI well-being OR AB well-being) OR (TI wellness OR AB wellness) OR (TI "standard

					of living" OR AB "standard of living") OR (TI "daily functioning" OR AB "daily functioning") OR (TI "routine activities" OR AB "routine activities") OR (TI "social functioning" OR AB "social functioning") OR (TI "functional status" OR AB "functional status") OR (TI HiSQOL OR AB HiSQOL) OR (TI DLQI OR AB DLQI) OR (TI Skindex OR AB Skindex) OR (TI EQ-5D OR AB EQ-5D) OR (TI SF-36 OR AB SF-36)) AND<br>((TI subjective OR AB subjective) OR (TI self-reported OR AB self-reported) OR (TI patient-reported OR AB patient-reported))
**Web of Science Core Collection**	Clarivate	18 March 2026	None	158	("Hidradenitis Suppurativa" OR "hidradenitis suppurativa" OR "acne inversa") AND ("Quality of Life" OR "quality of life" OR QoL OR HRQoL OR "health-related quality of life" OR "life satisfaction" OR "life quality" OR well-being OR wellness OR "standard of living" OR "daily functioning" OR "routine activities" OR "social functioning" OR "functional status" OR HiSQOL OR DLQI OR Skindex OR EQ-5D OR SF-36) AND<br>(subjective OR self-reported OR patient-reported)
**Scopus**	Elsevier	18 March 2026	None	34	("Hidradenitis Suppurativa" OR "hidradenitis suppurativa" OR "acne inversa") AND<br>("Quality of Life" OR "quality of life" OR QoL OR HRQoL OR "health-related quality of life" OR "life satisfaction" OR "life quality" OR well-being OR wellness OR "standard of living" OR "daily functioning" OR "routine activities" OR "social functioning" OR "functional status" OR HiSQOL OR DLQI OR Skindex OR EQ-5D OR SF-36) AND<br>(subjective OR self-reported OR patient-reported)
**PsycINFO**	Ovid	18 March 2026	None	24	(exp "Hidradenitis Suppurativa"/ OR "hidradenitis suppurativa".ti,ab. OR "acne inversa".ti,ab.) AND<br>(exp "Quality of Life"/ OR "quality of life".ti,ab. OR QoL.ti,ab. OR HRQoL.ti,ab. OR "health-related quality of life".ti,ab. OR "life satisfaction".ti,ab. OR "life quality".ti,ab. OR well-being.ti,ab. OR wellness.ti,ab. OR "standard of living".ti,ab. OR "daily functioning".ti,ab. OR "routine activities".ti,ab. OR "social functioning".ti,ab. OR "functional status".ti,ab. OR HiSQOL.ti,ab. OR DLQI.ti,ab. OR Skindex.ti,ab. OR EQ-5D.ti,ab. OR SF-36.ti,ab.) AND<br>(subjective.ti,ab. OR self-reported.ti,ab. OR patient-reported.ti,ab.)
