# Supplementary material for: Quality of Life in Patients with Hidradenitis Suppurativa: A Scoping Review
Source: Nurs Rep. 2026 Aug 19;16(8):289. doi: 10.3390/nursrep16080289 (PMC13516480; doi:10.3390/nursrep16080289)
Supplement: Supplementary file 1 [file nursrep-16-00289-s001.zip › Supplementary Table S3.pdf]

Supplementary Table S3: Characteristics of the included primary studies

Author	Year	Country	Study design	Sample size (n)	Key population characteristics	Objective	Main QoL/PRO findings
**Kaaz et al.**	2018	Poland	Case–control observational study	**108 HS + 50 controls**	HS patients: 51 females and 57 males; mean age 36.3 ± 12.1 years; mean disease duration 9.1 ± 8.3 years. Patients had clinically diagnosed HS, with disease severity assessed using HSS, HSSI and Hurley stage. Pain and pruritus were assessed as key HS-related symptoms.	To evaluate the influence of pain and pruritus on sleep quality in patients with HS.	Pain and pruritus were associated with poorer sleep quality. Pain had a particularly relevant effect on subjective sleep quality, sleep duration, and daytime dysfunction.
**Frings et al.**	2019	Germany	Cross-sectional observational study	**110**	Mean age 38 ± 12 years (range 18–75); 61 females (55%) and 49 males (45%). Mean BMI was 30.5 ± 6.9 kg/m²; 49% had BMI ≥30 kg/m². Regarding disease severity, 7% had Hurley stage I, 30% stage II and 63% stage III. Approximately 37% were current smokers.	To assess the psychological burden of HS, including anxiety, depression, quality of life and psychosocial impairment.	QoL was substantially impaired, with a mean DLQI of 12 ± 7. Skindex-29 scores indicated marked impairment in emotional and functional domains. Anxiety and depressive symptoms were also frequent, and pain was significantly associated with poorer QoL and psychological burden.
**Quinto et al.**	2021	Italy	Cross-sectional study	**77**	Patients with HS; the study collected sociodemographic, lifestyle, clinical and psychological characteristics. Participants were assessed for sexual desire and functioning, QoL, anxiety, depression and minor	To assess sexual desire and sexual functioning impairment in patients with HS and their associations with QoL, psychologic	The majority of patients reported hindered sexuality and impaired sexual functioning, despite relatively preserved dyadic and solitary sexual

					psychiatric disorders, as well as disease-related impairment of sexuality.	al burden and clinical characteristics	desire. Sexual impairment was examined in relation to QoL and psychological burden.
**Kokolakis et al.**	2025	Multinational (US, UK, Germany, France, Italy, Spain)	Cross-sectional online survey	**656** (505 diagnosed HS; 151 suspected HS)	Adults with self-reported diagnosed or suspected HS. Mean age 41 ± 11 years; 65% female. Among participants with available ethnicity data (n=456), 80% were White, 10% Hispanic, 7% Black and 2% Asian. Common symptoms included abscesses (84%), nodules/boils (75%) and tunnels (52%); the groin (66%) and armpits (65%) were the most frequently affected sites.	To investigate patient activation, the patient journey and the burden of HS, including QoL, mental health, work productivity and treatment priorities.	A very large and extremely large effect of HS on QoL was reported by 41% and 28% of participants, respectively. Among diagnosed patients, abnormal anxiety and depression were reported by 53% and 29%, respectively, while overall work impairment was 52% among employed participants.
**Schneider-Burrus et al.**	2023	Germany	Prospective multicentre epidemiological non-interventional study	**481**	Mean age 39.6 ± 11.6 years; 53% female. Mean BMI 31.1 ± 7.0 kg/m². Mean disease duration 14.6 ± 10.5 years and mean age at first symptoms 24.8 ± 10.9 years. Mean Hurley score 2.0 ± 0.7; 24.0% Hurley I, 49.3% Hurley II and 26.7% Hurley III. Approximately 62.3% were current smokers and 23.7% former smokers.	To determine the extent of work ability and productivity impairment associated with HS in Germany.	HS was associated with substantial impairment in professional functioning. Among employed patients, HS-related absenteeism was 13.3%, presenteeism was 25.2%, and overall work impairment was 33.4%; presenteeism was associated with disease severity.

**Schneider-Burrus et al.**	2021	Germany	Cross-sectional observational study	**462**	462 patients with available QoL data; 278 females and 184 males. Mean age approximately 40 years. Patients had clinically diagnosed HS and were assessed for demographic, anamnestic and clinical characteristics, including BMI, smoking, disease severity and comorbidities. QoL was assessed using the DLQI.	To identify demographic and clinical factors associated with QoL impairment in patients with HS.	Mean DLQI was 13.18 ± 7.99, indicating a profound impact of HS on QoL. Very large and extremely large QoL impairment were observed in 40.3% and 20.1% of patients, respectively. Symptoms, pain, and embarrassment /self-consciousness were among the most affected domains.
**Molina-Leyva et al.**	2020	Spain	Cross-sectional study	**233**	233 patients with HS. Pruritus and malodour were assessed using numerical rating scales and QoL using the DLQI. Pruritus severity was associated with the number of affected regions, female sex, intensity of suppuration and Crohn's disease; statin use was associated with lower pruritus intensity.	To investigate the impact of pruritus and malodour on QoL and their associations with clinical characteristics in patients with HS.	Mean DLQI was 13.99 ± 10.59. Both pruritus and malodour were positively associated with poorer QoL; pruritus severity was also associated with the number of affected body regions, female sex, suppuration intensity, and Crohn's disease.
**Alavi et al.**	2015	Canada	Prospective case series	**55**	38 females and 17 males with HS recruited from community dermatology clinics in Ontario. Disease severity was assessed using Hurley staging and lesion counts. QoL was assessed using	To assess QoL impairment in Canadian patients with HS and examine its relationship with disease severity.	Mean DLQI was 10 ± 8.8, indicating a moderate effect of HS on patients' lives. Hurley stage, lesion number, and patient-reported QoL were significantly

					the DLQI and SF-36v2.		correlated with DLQI scores.
**Matusiak et al.**	2010	Poland	Cross-sectional observational study	**54**	Patients with active but clinically stable HS, aged 16–65 years.	To assess the broad psychophysical burden of HS and its impact on QoL, depression, fatigue and health-related well-being.	HS had a substantial negative impact on patients' lives. Mean DLQI was 12.67 ± 7.7, with approximately 60% of patients reporting a large or extremely large effect of HS on their lives. Mean BDI-SF was 5.87 ± 4.68, with around 20% of patients showing scores suggestive of possible depression. Mean EQ-5D health index was 0.66 ± 0.23 and EQ-VAS 56.78 ± 18.84, indicating impaired perceived health. FACIT-F (32.06 ± 11.01) indicated relevant fatigue, while Q-LES-Q-SF (56.44 ± 15.17%) indicated reduced life satisfaction. Disease severity was the main factor associated with poorer psychophysical well-being, while anogenital involvement was particularly associated with impaired QoL.

**Alamri et al.**	2021	Saudi Arabia	Cross-sectional study	**36**	24 males (66.7%) and 12 females (33.3%); mean age 35.9 ± 10.7 years (range 18–65). Hurley stage I: 7 (21.1%); stage II: 9 (27.3%); stage III: 17 (51.5%); 3 patients were unstageable. The axillae were the most frequently affected anatomical sites.	To assess QoL in patients with HS and investigate its relationship with disease severity and anatomical distribution.	Mean DLQI was 15.3 ± 8.3, corresponding to a very large effect of HS on patients' lives. No statistically significant association was found between Hurley stage and DLQI in this small cohort.
**Kimball et al. / UNITE registry**	2020	Multinational (12 countries)	Prospective observational international multicentre registry	**594**	89.1% adults and 10.9% adolescents; 69.7% female and 81.2% White. Most patients had moderate-to-severe disease (Hurley II/III: 93.3%). Previous HS procedures/surgery had been performed in 68.7%; 61.6% of adults and 49.2% of adolescents were obese, 40.2% were current tobacco users and 91.2% had scarring.	To describe the baseline clinical characteristics, disease history and patient-reported outcomes of patients with HS enrolled in the international UNITE registry.	In adults, the mean DLQI was 12.6 ± 8.0, indicating a large effect of HS on QoL; in adolescents, the mean CDLQI was 6.9 ± 6.2. QoL impairment increased with disease severity. Patient-reported symptoms, embarrassment/self-consciousness, clothing choices, anxiety/depression, disease control, and work productivity were also affected.
**Krajewski et al.**	2021	Germany	Cross-sectional study	**1,795**	Mean age 40.0 ± 11.8 years; 1,152 females and 643 males. Mean BMI 28.1 ± 6.2 kg/m², indicating an overweight population on average. Approximately 55.6% were	To quantify QoL impairment in a large German HS cohort and investigate demographic and clinical factors	Mean DLQI was 13.2 ± 8.1, indicating substantial QoL impairment. Greater impairment was associated with female sex, pain, and

					smokers. Disease severity was assessed using Hurley staging and, in 634 patients, IHS4. Mean DLQI was 13.2 ± 8.1, with greater QoL impairment among females and increasing impairment associated with pain and disease severity.	associated with QoL impairment.	increasing disease severity.
**Andersen et al.**	2020	Denmark	Cross-sectional comparative study	**141 HS + 144 controls**	HS patients were recruited from a dermatological outpatient clinic in Denmark; 138 had active HS and 3 additional HS patients were identified among the control population and reclassified accordingly, resulting in 141 HS patients and 144 controls. The study population was assessed for body-image-related QoL, BMI, sex, age and depressive symptoms.	To compare body-image-related QoL in patients with HS with patients with other dermatological conditions and identify factors associated with impaired body image.	HS was associated with significantly more negative body-image-related QoL than other dermatological conditions, even after adjustment for BMI, sex, age, and depressive symptoms.
**Riis et al.**	2016	Denmark	Cross-sectional study	**421**	421 patients with HS assessed using the EQ-5D questionnaire. Pain, malodour and pruritus were specifically evaluated as disease-related symptoms. Health utility was compared with Danish population reference values.	To quantify health-related utility/disutility in patients with HS and investigate the associations of pain, malodour and pruritus	Mean EQ-5D index was 0.705 compared with 0.887 in the Danish reference population, while mean VAS was 62.25 compared with 82.6. Pain, malodour, and pruritus were significantly associated with

						with health utility.	reduced health utility.
**Matusiak et al.**	2010	Poland	Observational cross-sectional study; letter to the editor/report of original data	54 patients with HS; 30 patients were professionally active	Patients with hidradenitis suppurativa (HS), aged 16–65 years. Disease severity was assessed according to the Hurley classification.	To assess the impact of HS on quality of life (QoL) and p rofessional /work activity.	QoL was markedly impaired, with a mean DLQI of 12.67 ± 7.7. Greater QoL impairment was associated with higher Hurley stage, a greater number of affected areas, and anogenital involvement. Among professionally active patients, 58.1% reported work absences attributable to HS, with an average of approximately 3 4 days of absence per year. 10%had lost their job because of HS-related difficulties, while 23.3% rep orted impaired professional advancement.
