# Supplementary material for: Quality of Life in Patients with Hidradenitis Suppurativa: A Scoping Review
Source: Nurs Rep. 2026 Aug 19;16(8):289. doi: 10.3390/nursrep16080289 (PMC13516480; doi:10.3390/nursrep16080289)
Supplement: Supplementary file 1 [file nursrep-16-00289-s001.zip › Supplementary Table S4.pdf]

Supplementary Table S4: Full-Text screening exclusions

N	References	Reason of exclusion
**1**	Ingram JR, Woo PN, Chua SL, Ormerod AD, Desai N, Kai AC, et al. – *Interventions for hidradenitis suppurativa*	Intervention/treatment-focused; does not map QoL/HRQoL as the primary concept of the review.
**2**	Chernyshov PV, Zouboulis CC, Tomas-Aragones L, et al. – *Quality of life measurement in hidradenitis suppurativa: position statement of the European Academy of Dermatology and Venereology task forces on Quality of Life and Patient-Oriented Outcomes and Acne, Rosacea and Hidradenitis Suppurativa*	Post-treatment context not within the scope of the review.
**3**	Kirby JS, Hereford B, Thorlacius L, Villumsen B, Ingram JR, Garg A, et al. – *Validation of global item for assessing impact on quality of life of patients with hidradenitis suppurativa*	Validation of a QoL assessment item rather than mapping QoL outcomes.
**4**	Thorlacius L, Esmann S, Miller I, Vinding G, Jemec GBE. – *Development of HiSQOL: A Hidradenitis Suppurativa-Specific Quality of Life Instrument*	Exclusively focused on development and pilot testing of a QoL instrument.
**5**	Chodziuk A, Holgersen N, Nielsen VW, Thyssen JP, Egeberg A, Thomsen SF. – *Fatigue is associated with disease severity in adult patients with hidradenitis suppurativa*	Primary focus on fatigue rather than mapping QoL/HRQoL.
**6**	Rondags A, van Straalen KR, van Hasselt JR, Janse IC, Ardon CB, Vossen ARJV, Prens EP, van der Zee HH, Horváth B. – *Correlation of the refined Hurley classification for hidradenitis suppurativa with patient-reported quality of life and objective disease severity assessment*	Primary focus on disease severity rather than mapping QoL.
**7**	Nielsen RM, Lindsø Andersen P, Sigsgaard V, Theut Riis P, Jemec	Primary focus on pain rather than mapping QoL/HRQoL.

	GB. – *Pain perception in patients with hidradenitis suppurativa*	
**8**	Greene A, Hwang AS, Amjad S, Kechter JA, Mangold AR, Chen SX. – *Concordance of DLQI and pain score in hidradenitis suppurativa clinical trials: a systematic review*	Focus on clinical trial outcomes and concordance of PROs rather than mapping QoL/HRQoL in patients with HS.
**9**	Li YH, Speck P, Ingram JR, Orenstein LAV. – *Comparing maximum and average numerical rating scale pain scores in hidradenitis suppurativa*	Primary focus on pain measurement rather than mapping QoL/HRQoL.
**10**	Jain S, Tushe S, Sood A, Yeung H, Orenstein LAV. – *Hidradenitis suppurativa and psychosocial well-being: a cross-sectional survey analysis from the All of Us research program*	Primary focus on psychosocial well-being rather than mapping QoL/HRQoL.
**11**	Erol Mart HM, Bostanci S, Caliskan Odabas D. – *Demographic and Clinical Features and Factors Affecting the Quality of Life of Patients With Hidradenitis Suppurativa: A Cross-sectional Study*	Study objective not aligned with the review objective
**12**	Macklis PC, Tyler K, Kaffenberger J, Kwatra S, Kaffenberger BH. – *Lifestyle modifications associated with symptom improvement in hidradenitis suppurativa patients*	Primary focus on symptom improvement and lifestyle modifications rather than mapping QoL/HRQoL
**13**	Ravn Jørgensen AH, Yao Y, Thomsen SF, Christian Ring H. – *Self-Reported Pain Alleviating Methods in Patients with Hidradenitis Suppurativa*	Primary focus on pain and pain-alleviating methods rather than mapping QoL/HRQoL.
**14**	Kaaz K, Szepietowski JC, Matusiak Ł. – *Sleep quality among adult patients with chronic dermatoses*	Primary focus on sleep quality rather than mapping QoL/HRQoL in HS.
**15**	Otten M, Augustin M, Blome C, Topp J, Niklaus M, Hilbring C, Bechara FG, Pinter A, Zouboulis CC, Anzengruber F, Kirsten N. – *Measuring Quality of Life in*	Exclusively focused on development and validation of a QoL instrument.

	Hidradenitis Suppurativa: Development and Validation of a Disease-specific Patient-reported Outcome Measure for Practice and Research	
**16**	Kimball AB, Kirby JS, Ingram JR, Tran T, Pansar I, Ciaravino V, Willems D, Lewis-Mikhael AM, Garg A. – *A systematic literature review of health-related quality of life and other patient reported outcomes across key subgroups in hidradenitis suppurativa*	Systematic literature review focused on subgroup analysis rather than direct mapping of QoL in the target population.
**17**	Gergely LH, Gáspár K, Brodszky V, Kinyó Á, Szegedi A, Remenyik É, Kiss NF, Bató A, Péntek M, Gulácsi L, Sárdy M, Bánvölgyi A, Wikonkál N, Rencz F. – *Validity of EQ-5D-5L, Skindex-16, DLQI and DLQI-R in patients with hidradenitis suppurativa*	Exclusively focused on validation of QoL/HRQoL instruments.
**18**	Marzano AV, Chiricozzi A, Giovanardi G, Argenziano G, Bettoli V, Bianchi L, Dapavo P, Dini V, Fabbrocini G, Micali G, Offidani AM, Potenza C, Prignano F, Gualberti G, Saragaglia V, Rongioletti F. – *Creation of a severity index for hidradenitis suppurativa that includes a validated quality-of-life measure: the HIDRAscore*	Primary focus on development of a disease-severity index rather than mapping QoL/HRQoL.
**19**	Maghfour J, Sivesind TE, Dellavalle RP, Dunnick C. – *Trends in Hidradenitis Suppurativa Disease Severity and Quality of Life Outcome Measures: Scoping Review*	Focus on QoL/severity outcome measures used in clinical trials rather than mapping QoL in patients with HS.
**20**	Garg A, Burge R, Cohee A, Wallinger H, Truman I, Keal A, Strunk A, Barlow S. – *Validation of the real-world application of the Hidradenitis Suppurativa Quality of Life (HiSQOL) score to adults with hidradenitis suppurativa*	Exclusively focused on validation of a QoL instrument.

**21**	Bailey SL, Buckingham C, Maxwell-Scott H, Rashidghamat E, Ferguson FJ, Malhomme de la Roche H, Goldenberg SD. – *Microbial Isolates and Association with Disease Severity and Quality of Life in Individuals with Hidradenitis Suppurativa: An Observational Study*	Primary focus on microbial isolates and disease severity rather than mapping QoL/HRQoL.
